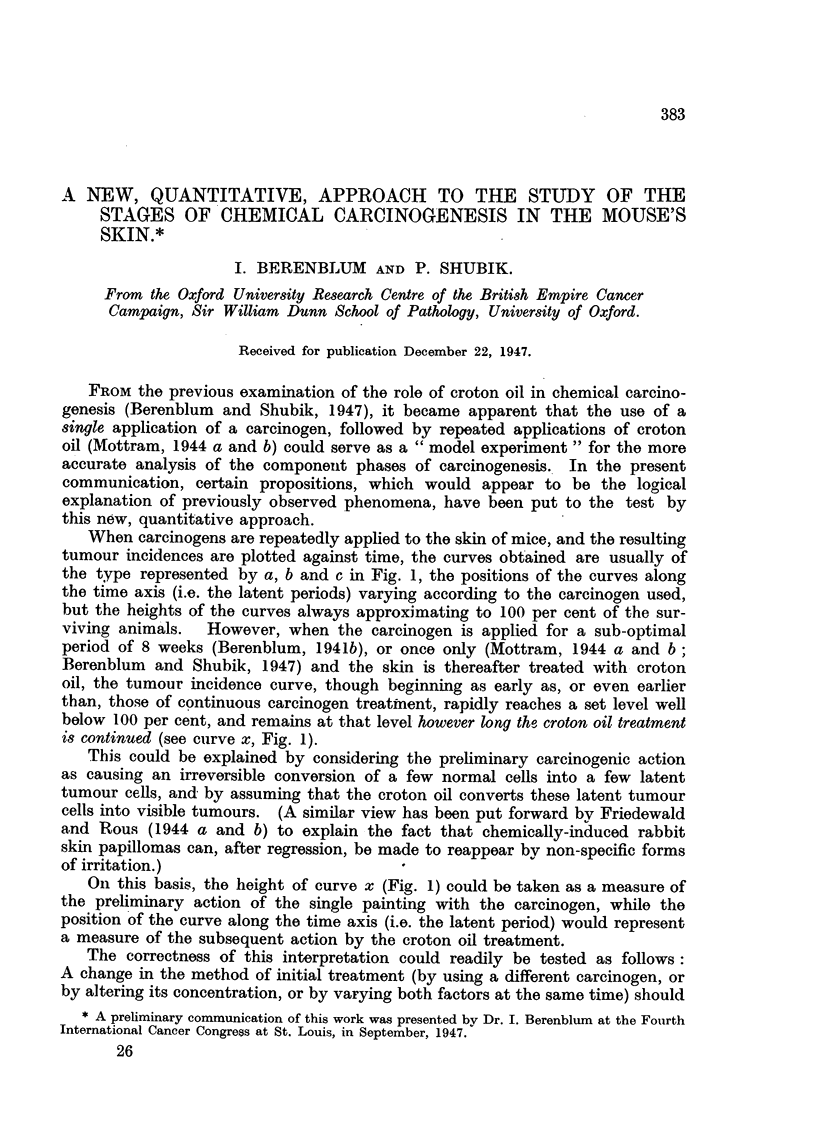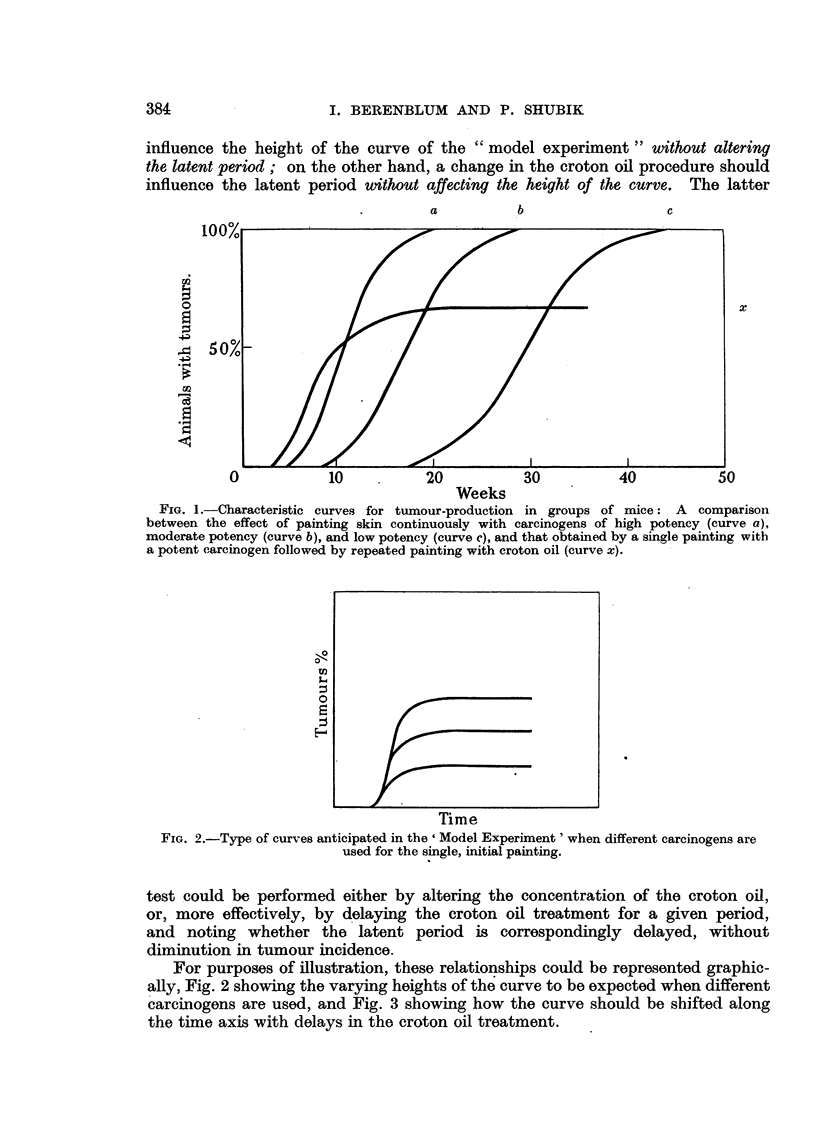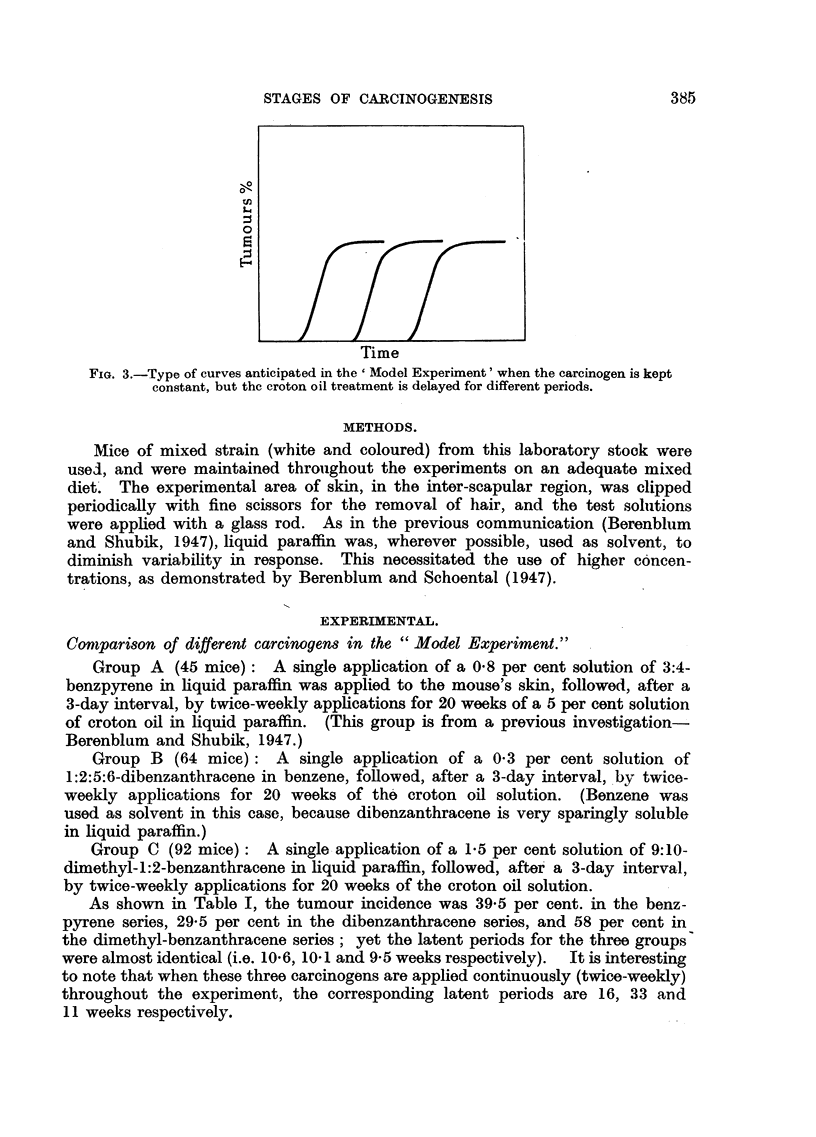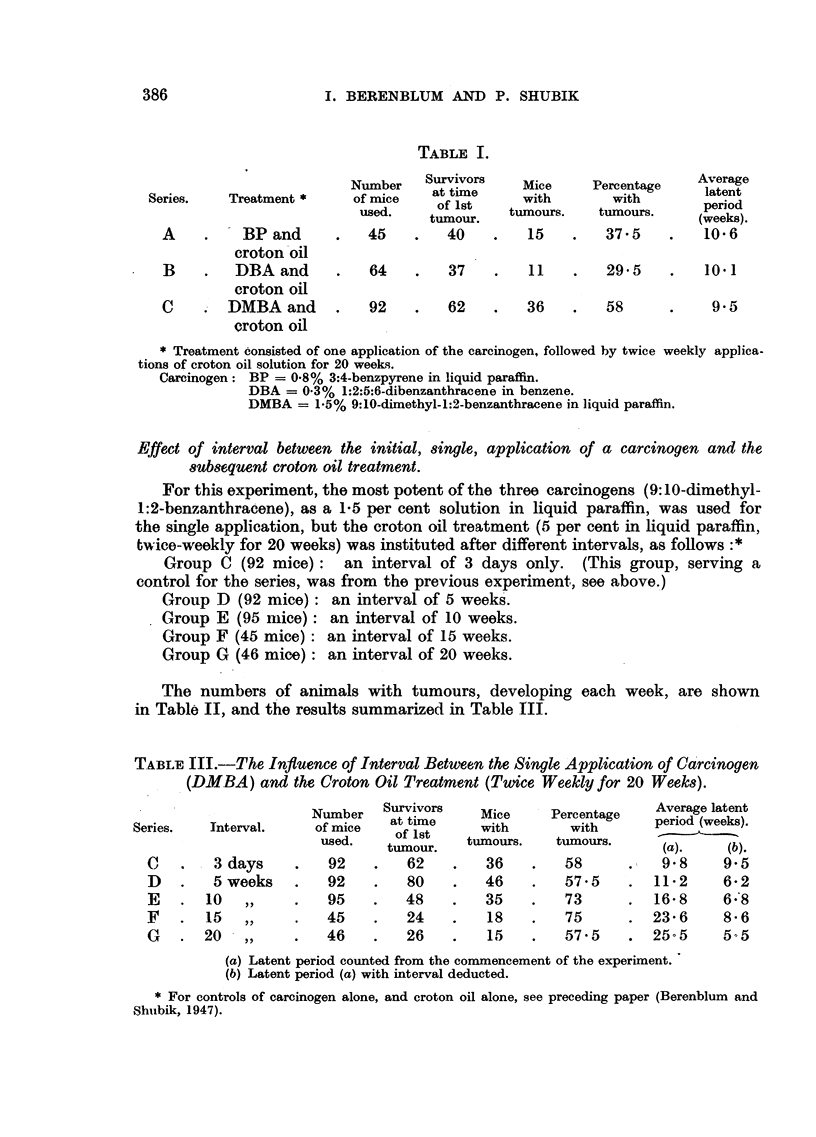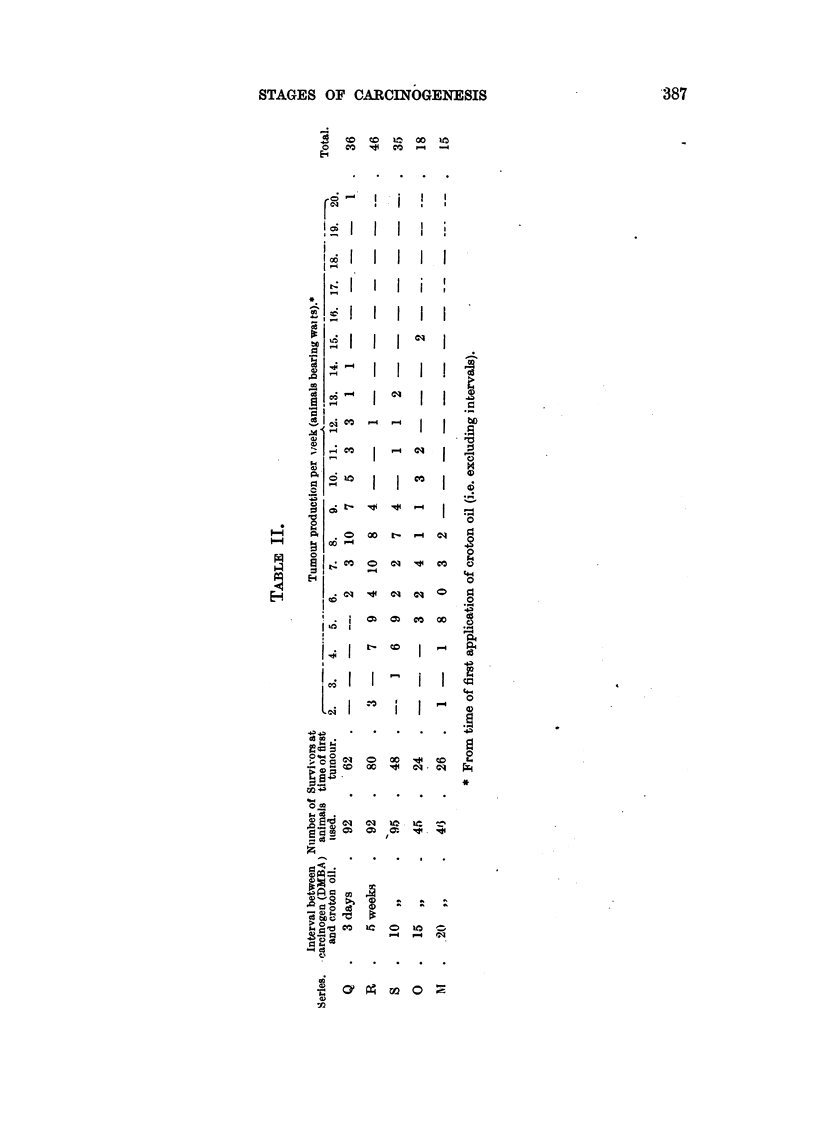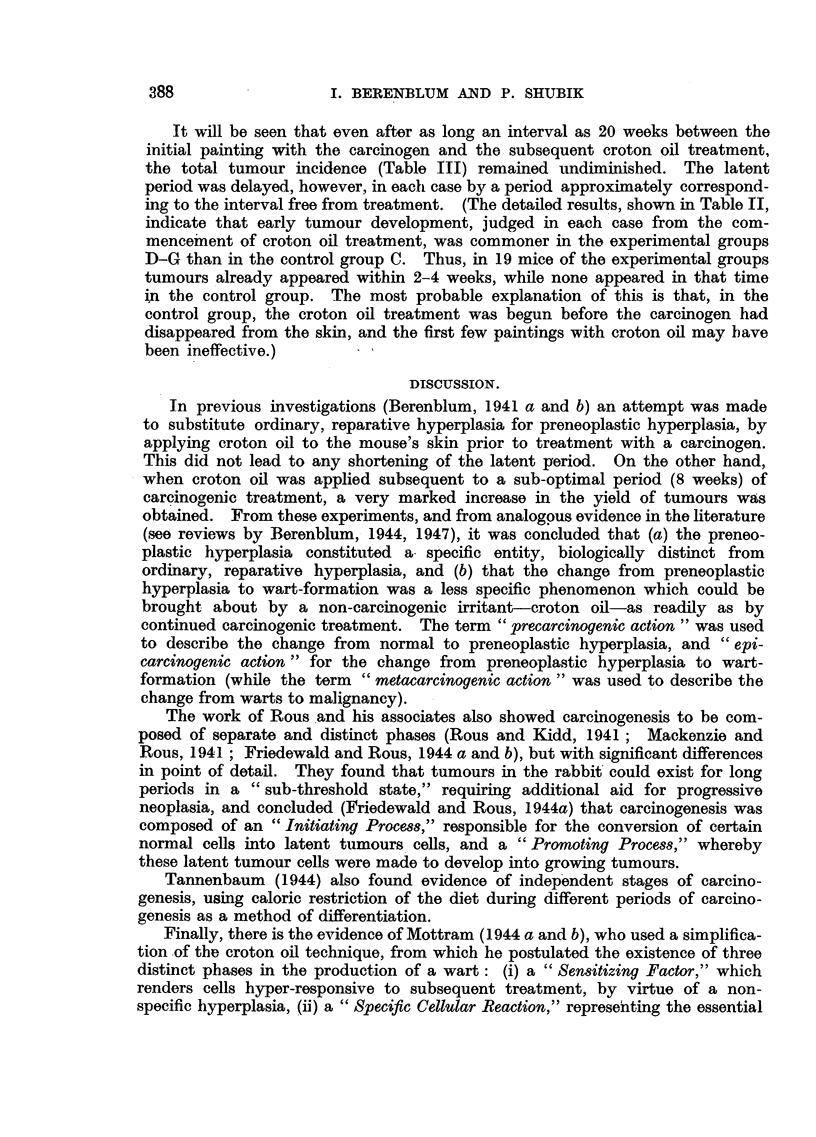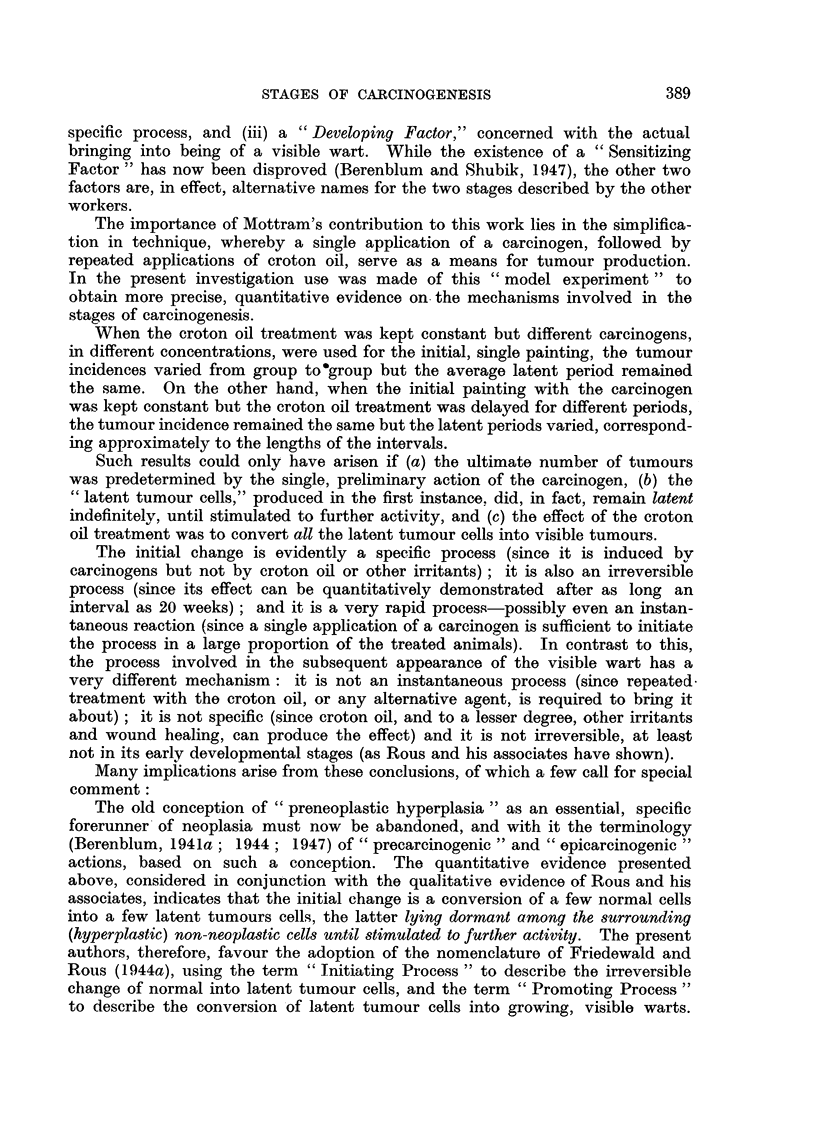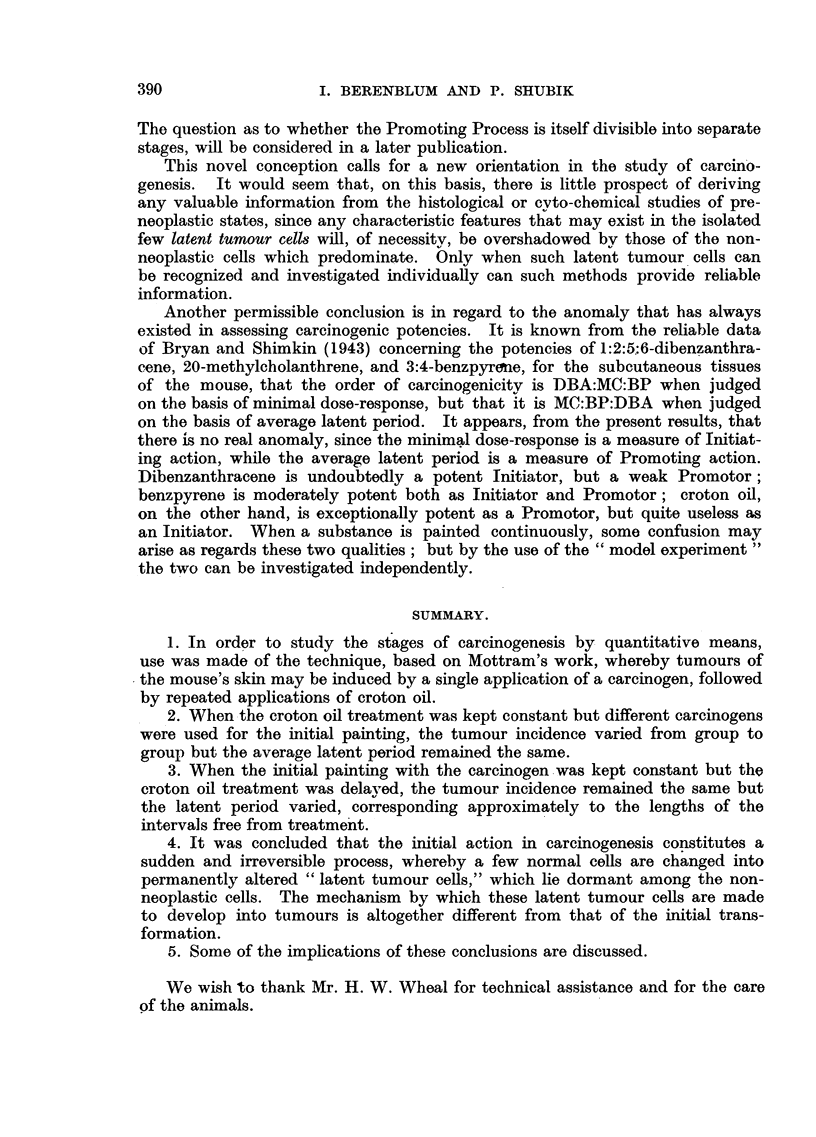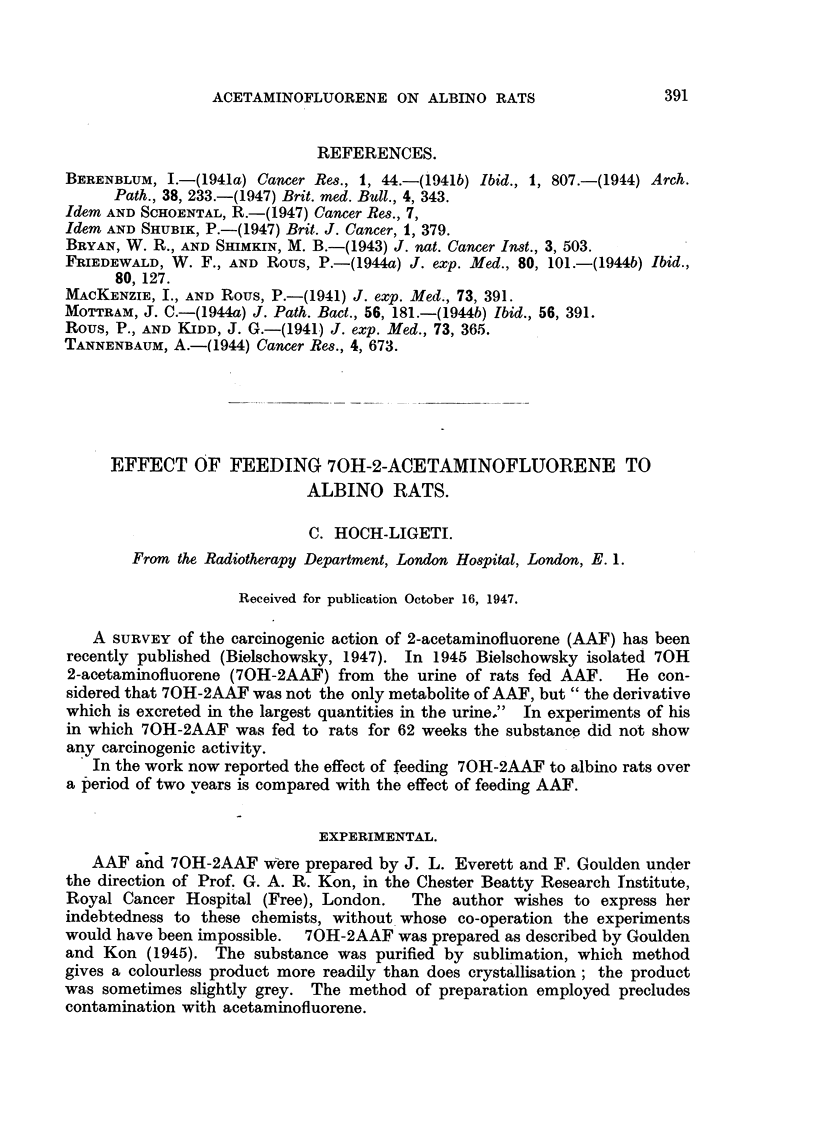# A New, Quantitative, Approach to the Study of the Stages of Chemical Carcinogenesis in the Mouse's Skin*

**DOI:** 10.1038/bjc.1947.36

**Published:** 1947-12

**Authors:** I. Berenblum, P. Shubik


### A NEW, QUANTITATIVE, APPROACH TO THE STUDY OF THE STAGES OF CHEMICAL CARCINOGENESIS IN THE MOUSE'S SKIN.*

I. BERENBLUM AND P. SHUBIK.

*From the Oxford University Research Centre of the British Empire Cancer Campaign, Sir William Dunn School of Pathology, University of Oxford.*

Received for publication December 22, 1947.

FROM the previous examination of the role of croton oil in chemical carcinogenesis (Berenblum and Shubik, 1947), it became apparent that the use of a *single* application of a carcinogen, followed by repeated applications of croton oil (Mottram, 1944 *a* and *b*) could serve as a " model experiment " for the more accurate analysis of the component phases of carcinogenesis. In the present communication, certain propositions, which would appear to be the logical explanation of previously observed phenomena, have been put to the test by this new, quantitative approach.

When carcinogens are repeatedly applied to the skin of mice, and the resulting tumour incidences are plotted against time, the curves obtained are usually of the type represented by *a*, *b* and *c* in Fig. 1, the positions of the curves along the time axis (i.e. the latent periods) varying according to the carcinogen used, but the heights of the curves always approximating to 100 per cent of the surviving animals. However, when the carcinogen is applied for a sub-optimal period of 8 weeks (Berenblum, 1941*b*), or once only (Mottram, 1944 *a* and *b* ; Berenblum and Shubik, 1947) and the skin is thereafter treated with croton oil, the tumour incidence curve, though beginning as early as, or even earlier than, those of continuous carcinogen treatment, rapidly reaches a set level well below 100 per cent, and remains at that level *however long the croton oil treatment is continued* (see curve *x*, Fig. 1).

This could be explained by considering the preliminary carcinogenic action as causing an irreversible conversion of a few normal cells into a few latent tumour cells, and by assuming that the croton oil converts these latent tumour cells into visible tumours. (A similar view has been put forward by Friedewald and Rous (1944 *a* and *b*) to explain the fact that chemically-induced rabbit skin papillomas can, after regression, be made to reappear by non-specific forms of irritation.)

On this basis, the height of curve *x* (Fig. 1) could be taken as a measure of the preliminary action of the single painting with the carcinogen, while the position of the curve along the time axis (i.e. the latent period) would represent a measure of the subsequent action by the croton oil treatment.

The correctness of this interpretation could readily be tested as follows : A change in the method of initial treatment (by using a different carcinogen, or by altering its concentration, or by varying both factors at the same time) should

* A preliminary communication of this work was presented by Dr. I. Berenblum at the Fourth International Cancer Congress at St. Louis, in September, 1947.

influence the height of the curve of the " model experiment " *without altering the latent period ;* on the other hand, a change in the croton oil procedure should influence the latent period *without affecting the height of the curve.* The latter

FIG. 1.—Characteristic curves for tumour-production in groups of mice: A comparison between the effect of painting skin continuously with carcinogens of high potency (curve *a*), moderate potency (curve *b*), and low potency (curve *c*), and that obtained by a single painting with a potent carcinogen followed by repeated painting with croton oil (curve *x*).

FIG. 2.—Type of curves anticipated in the ' Model Experiment ' when different carcinogens are used for the single, initial painting.

test could be performed either by altering the concentration of the croton oil, or, more effectively, by delaying the croton oil treatment for a given period, and noting whether the latent period is correspondingly delayed, without diminution in tumour incidence.

For purposes of illustration, these relationships could be represented graphically, Fig. 2 showing the varying heights of the curve to be expected when different carcinogens are used, and Fig. 3 showing how the curve should be shifted along the time axis with delays in the croton oil treatment.

FIG. 3.—Type of curves anticipated in the ' Model Experiment ' when the carcinogen is kept constant, but thc croton oil treatment is delayed for different periods.

### METHODS.

Mice of mixed strain (white and coloured) from this laboratory stock were used, and were maintained throughout the experiments on an adequate mixed diet. The experimental area of skin, in the inter-scapular region, was clipped periodically with fine scissors for the removal of hair, and the test solutions were applied with a glass rod. As in the previous communication (Berenblum and Shubik, 1947), liquid paraffin was, wherever possible, used as solvent, to diminish variability in response. This necessitated the use of higher concentrations, as demonstrated by Berenblum and Schoental (1947).

### EXPERIMENTAL.

*Comparison of different carcinogens in the " Model Experiment."*

Group A (45 mice) : A single application of a 0·8 per cent solution of 3:4-benzpyrene in liquid paraffin was applied to the mouse's skin, followed, after a 3-day interval, by twice-weekly applications for 20 weeks of a 5 per cent solution of croton oil in liquid paraffin. (This group is from a previous investigation—Berenblum and Shubik, 1947.)

Group B (64 mice) : A single application of a 0·3 per cent solution of 1:2:5:6-dibenzanthracene in benzene, followed, after a 3-day interval, by twice-weekly applications for 20 weeks of the croton oil solution. (Benzene was used as solvent in this case, because dibenzanthracene is very sparingly soluble in liquid paraffin.)

Group C (92 mice) : A single application of a 1·5 per cent solution of 9:10-dimethyl-1:2-benzanthracene in liquid paraffin, followed, after a 3-day interval, by twice-weekly applications for 20 weeks of the croton oil solution.

As shown in Table I, the tumour incidence was 39·5 per cent. in the benzpyrene series, 29·5 per cent in the dibenzanthracene series, and 58 per cent in the dimethyl-benzanthracene series ; yet the latent periods for the three groups were almost identical (i.e. 10·6, 10·1 and 9·5 weeks respectively). It is interesting to note that when these three carcinogens are applied continuously (twice-weekly) throughout the experiment, the corresponding latent periods are 16, 33 and 11 weeks respectively.

TABLE I.

| Series. | Treatment * | Number of mice used. | Survivors at time of 1st tumour. | Mice with tumours. | Percentage with tumours. | Average latent period (weeks). |
|---|---|---|---|---|---|---|
| A | BP and croton oil | 45 | 40 | 15 | 37·5 | 10·6 |
| B | DBA and croton oil | 64 | 37 | 11 | 29·5 | 10·1 |
| C | DMBA and croton oil | 92 | 62 | 36 | 58 | 9·5 |

* Treatment consisted of one application of the carcinogen, followed by twice weekly applications of croton oil solution for 20 weeks.

Carcinogen : BP = 0·8% 3:4-benzpyrene in liquid paraffin.
DBA = 0·3% 1:2:5:6-dibenzanthracene in benzene.
DMBA = 1·5% 9:10-dimethyl-1:2-benzanthracene in liquid paraffin.

*Effect of interval between the initial, single, application of a carcinogen and the subsequent croton oil treatment.*

For this experiment, the most potent of the three carcinogens (9:10-dimethyl-1:2-benzanthracene), as a 1·5 per cent solution in liquid paraffin, was used for the single application, but the croton oil treatment (5 per cent in liquid paraffin, twice-weekly for 20 weeks) was instituted after different intervals, as follows :*

Group C (92 mice) : an interval of 3 days only. (This group, serving a control for the series, was from the previous experiment, see above.)

Group D (92 mice) : an interval of 5 weeks.

Group E (95 mice) : an interval of 10 weeks.

Group F (45 mice) : an interval of 15 weeks.

Group G (46 mice) : an interval of 20 weeks.

The numbers of animals with tumours, developing each week, are shown in Table II, and the results summarized in Table III.

TABLE III.—*The Influence of Interval Between the Single Application of Carcinogen (DMBA) and the Croton Oil Treatment (Twice Weekly for 20 Weeks).*

| Series. | Interval. | Number of mice used. | Survivors at time of 1st tumour. | Mice with tumours. | Percentage with tumours. | Average latent period (weeks). (a). | (b). |
|---|---|---|---|---|---|---|---|
| C | 3 days | 92 | 62 | 36 | 58 | 9·8 | 9·5 |
| D | 5 weeks | 92 | 80 | 46 | 57·5 | 11·2 | 6·2 |
| E | 10 ,, | 95 | 48 | 35 | 73 | 16·8 | 6·8 |
| F | 15 ,, | 45 | 24 | 18 | 75 | 23·6 | 8·6 |
| G | 20 ,, | 46 | 26 | 15 | 57·5 | 25·5 | 5·5 |

(*a*) Latent period counted from the commencement of the experiment.
(*b*) Latent period (*a*) with interval deducted.

* For controls of carcinogen alone, and croton oil alone, see preceding paper (Berenblum and Shubik, 1947).

TABLE II.

| Series. | Interval between carcinogen (DMBA) and croton oil. | Number of animals used. | Survivors at time of first tumour. | Tumour production per week (animals bearing warts).* | | | | | | | | | | | | | | | | | | | Total. |
|---|---|---|---|---|---|---|---|---|---|---|---|---|---|---|---|---|---|---|---|---|---|---|---|
| | | | | 2. | 3. | 4. | 5. | 6. | 7. | 8. | 9. | 10. | 11. | 12. | 13. | 14. | 15. | 16. | 17. | 18. | 19. | 20. | |
| Q | 3 days | 92 | 62 | — | — | — | — | 2 | 3 | 10 | 7 | 5 | 3 | 3 | 1 | 1 | — | — | — | — | — | 1 | 36 |
| R | 5 weeks | 92 | 80 | 3 | — | 7 | 9 | 4 | 10 | 8 | 4 | — | — | 1 | — | — | — | — | — | — | — | — | 46 |
| S | 10 ,, | 95 | 48 | — | 1 | 6 | 9 | 2 | 2 | 7 | 4 | — | 1 | 1 | 2 | — | — | — | — | — | — | — | 35 |
| O | 15 ,, | 45 | 24 | — | — | — | 3 | 2 | 4 | 1 | 1 | 3 | 2 | — | — | — | 2 | — | — | — | — | — | 18 |
| M | 20 ,, | 46 | 26 | 1 | — | 1 | 8 | 0 | 3 | 2 | — | — | — | — | — | — | — | — | — | — | — | — | 15 |

It will be seen that even after as long an interval as 20 weeks between the initial painting with the carcinogen and the subsequent croton oil treatment, the total tumour incidence (Table III) remained undiminished. The latent period was delayed, however, in each case by a period approximately corresponding to the interval free from treatment. (The detailed results, shown in Table II, indicate that early tumour development, judged in each case from the commencement of croton oil treatment, was commoner in the experimental groups D–G than in the control group C. Thus, in 19 mice of the experimental groups tumours already appeared within 2–4 weeks, while none appeared in that time in the control group. The most probable explanation of this is that, in the control group, the croton oil treatment was begun before the carcinogen had disappeared from the skin, and the first few paintings with croton oil may have been ineffective.)

### DISCUSSION.

In previous investigations (Berenblum, 1941 *a* and *b*) an attempt was made to substitute ordinary, reparative hyperplasia for preneoplastic hyperplasia, by applying croton oil to the mouse's skin prior to treatment with a carcinogen. This did not lead to any shortening of the latent period. On the other hand, when croton oil was applied subsequent to a sub-optimal period (8 weeks) of carcinogenic treatment, a very marked increase in the yield of tumours was obtained. From these experiments, and from analogous evidence in the literature (see reviews by Berenblum, 1944, 1947), it was concluded that (*a*) the preneoplastic hyperplasia constituted a specific entity, biologically distinct from ordinary, reparative hyperplasia, and (*b*) that the change from preneoplastic hyperplasia to wart-formation was a less specific phenomenon which could be brought about by a non-carcinogenic irritant—croton oil—as readily as by continued carcinogenic treatment. The term "*precarcinogenic action*" was used to describe the change from normal to preneoplastic hyperplasia, and "*epicarcinogenic action*" for the change from preneoplastic hyperplasia to wart-formation (while the term "*metacarcinogenic action*" was used to describe the change from warts to malignancy).

The work of Rous and his associates also showed carcinogenesis to be composed of separate and distinct phases (Rous and Kidd, 1941; Mackenzie and Rous, 1941; Friedewald and Rous, 1944 *a* and *b*), but with significant differences in point of detail. They found that tumours in the rabbit could exist for long periods in a "sub-threshold state," requiring additional aid for progressive neoplasia, and concluded (Friedewald and Rous, 1944*a*) that carcinogenesis was composed of an "*Initiating Process*," responsible for the conversion of certain normal cells into latent tumours cells, and a "*Promoting Process*," whereby these latent tumour cells were made to develop into growing tumours.

Tannenbaum (1944) also found evidence of independent stages of carcinogenesis, using caloric restriction of the diet during different periods of carcinogenesis as a method of differentiation.

Finally, there is the evidence of Mottram (1944 *a* and *b*), who used a simplification of the croton oil technique, from which he postulated the existence of three distinct phases in the production of a wart: (i) a "*Sensitizing Factor*," which renders cells hyper-responsive to subsequent treatment, by virtue of a nonspecific hyperplasia, (ii) a "*Specific Cellular Reaction*," representing the essential specific process, and (iii) a "*Developing Factor*," concerned with the actual bringing into being of a visible wart. While the existence of a "Sensitizing Factor" has now been disproved (Berenblum and Shubik, 1947), the other two factors are, in effect, alternative names for the two stages described by the other workers.

The importance of Mottram's contribution to this work lies in the simplification in technique, whereby a single application of a carcinogen, followed by repeated applications of croton oil, serve as a means for tumour production. In the present investigation use was made of this "model experiment" to obtain more precise, quantitative evidence on the mechanisms involved in the stages of carcinogenesis.

When the croton oil treatment was kept constant but different carcinogens, in different concentrations, were used for the initial, single painting, the tumour incidences varied from group to group but the average latent period remained the same. On the other hand, when the initial painting with the carcinogen was kept constant but the croton oil treatment was delayed for different periods, the tumour incidence remained the same but the latent periods varied, corresponding approximately to the lengths of the intervals.

Such results could only have arisen if (*a*) the ultimate number of tumours was predetermined by the single, preliminary action of the carcinogen, (*b*) the "latent tumour cells," produced in the first instance, did, in fact, remain *latent* indefinitely, until stimulated to further activity, and (*c*) the effect of the croton oil treatment was to convert *all* the latent tumour cells into visible tumours.

The initial change is evidently a specific process (since it is induced by carcinogens but not by croton oil or other irritants); it is also an irreversible process (since its effect can be quantitatively demonstrated after as long an interval as 20 weeks); and it is a very rapid process—possibly even an instantaneous reaction (since a single application of a carcinogen is sufficient to initiate the process in a large proportion of the treated animals). In contrast to this, the process involved in the subsequent appearance of the visible wart has a very different mechanism: it is not an instantaneous process (since repeated treatment with the croton oil, or any alternative agent, is required to bring it about); it is not specific (since croton oil, and to a lesser degree, other irritants and wound healing, can produce the effect) and it is not irreversible, at least not in its early developmental stages (as Rous and his associates have shown).

Many implications arise from these conclusions, of which a few call for special comment:

The old conception of "preneoplastic hyperplasia" as an essential, specific forerunner of neoplasia must now be abandoned, and with it the terminology (Berenblum, 1941*a*; 1944; 1947) of "precarcinogenic" and "epicarcinogenic" actions, based on such a conception. The quantitative evidence presented above, considered in conjunction with the qualitative evidence of Rous and his associates, indicates that the initial change is a conversion of a few normal cells into a few latent tumours cells, the latter *lying dormant among the surrounding (hyperplastic) non-neoplastic cells until stimulated to further activity.* The present authors, therefore, favour the adoption of the nomenclature of Friedewald and Rous (1944*a*), using the term "Initiating Process" to describe the irreversible change of normal into latent tumour cells, and the term "Promoting Process" to describe the conversion of latent tumour cells into growing, visible warts.

The question as to whether the Promoting Process is itself divisible into separate stages, will be considered in a later publication.

This novel conception calls for a new orientation in the study of carcinogenesis. It would seem that, on this basis, there is little prospect of deriving any valuable information from the histological or cyto-chemical studies of pre-neoplastic states, since any characteristic features that may exist in the isolated few *latent tumour cells* will, of necessity, be overshadowed by those of the non-neoplastic cells which predominate. Only when such latent tumour cells can be recognized and investigated individually can such methods provide reliable information.

Another permissible conclusion is in regard to the anomaly that has always existed in assessing carcinogenic potencies. It is known from the reliable data of Bryan and Shimkin (1943) concerning the potencies of 1:2:5:6-dibenzanthracene, 20-methylcholanthrene, and 3:4-benzpyrene, for the subcutaneous tissues of the mouse, that the order of carcinogenicity is DBA:MC:BP when judged on the basis of minimal dose-response, but that it is MC:BP:DBA when judged on the basis of average latent period. It appears, from the present results, that there is no real anomaly, since the minimal dose-response is a measure of Initiating action, while the average latent period is a measure of Promoting action. Dibenzanthracene is undoubtedly a potent Initiator, but a weak Promotor; benzpyrene is moderately potent both as Initiator and Promotor; croton oil, on the other hand, is exceptionally potent as a Promotor, but quite useless as an Initiator. When a substance is painted continuously, some confusion may arise as regards these two qualities; but by the use of the " model experiment " the two can be investigated independently.

### SUMMARY.

1. In order to study the stages of carcinogenesis by quantitative means, use was made of the technique, based on Mottram's work, whereby tumours of the mouse's skin may be induced by a single application of a carcinogen, followed by repeated applications of croton oil.

2. When the croton oil treatment was kept constant but different carcinogens were used for the initial painting, the tumour incidence varied from group to group but the average latent period remained the same.

3. When the initial painting with the carcinogen was kept constant but the croton oil treatment was delayed, the tumour incidence remained the same but the latent period varied, corresponding approximately to the lengths of the intervals free from treatment.

4. It was concluded that the initial action in carcinogenesis constitutes a sudden and irreversible process, whereby a few normal cells are changed into permanently altered " latent tumour cells," which lie dormant among the non-neoplastic cells. The mechanism by which these latent tumour cells are made to develop into tumours is altogether different from that of the initial transformation.

5. Some of the implications of these conclusions are discussed.

We wish to thank Mr. H. W. Wheal for technical assistance and for the care of the animals.

### REFERENCES.

BERENBLUM, I.—(1941*a*) *Cancer Res.*, **1**, 44.—(1941*b*) *Ibid.*, **1**, 807.—(1944) *Arch. Path.*, **38**, 233.—(1947) *Brit. med. Bull.*, **4**, 343.
*Idem* AND SCHOENTAL, R.—(1947) *Cancer Res.*, **7**,
*Idem* AND SHUBIK, P.—(1947) *Brit. J. Cancer*, **1**, 379.
BRYAN, W. R., AND SHIMKIN, M. B.—(1943) *J. nat. Cancer Inst.*, **3**, 503.
FRIEDEWALD, W. F., AND ROUS, P.—(1944*a*) *J. exp. Med.*, **80**, 101.—(1944*b*) *Ibid.*, **80**, 127.
MACKENZIE, I., AND ROUS, P.—(1941) *J. exp. Med.*, **73**, 391.
MOTTRAM, J. C.—(1944*a*) *J. Path. Bact.*, **56**, 181.—(1944*b*) *Ibid.*, **56**, 391.
ROUS, P., AND KIDD, J. G.—(1941) *J. exp. Med.*, **73**, 365.
TANNENBAUM, A.—(1944) *Cancer Res.*, **4**, 673.

---